# State-dependent metabolic partitioning and energy conservation: A theoretical framework for understanding the function of sleep

**DOI:** 10.1371/journal.pone.0185746

**Published:** 2017-10-10

**Authors:** Markus H. Schmidt, Theodore W. Swang, Ian M. Hamilton, Janet A. Best

**Affiliations:** 1 Department of Neurology, University of Bern, Inselspital, Bern, Switzerland; 2 Ohio Sleep Medicine and Neuroscience Institute, Dublin, Ohio, United States of America; 3 Department of Mathematics, The Ohio State University, Columbus, Ohio, United States of America; 4 Department of Evolution, Ecology and Organismal Biology, The Ohio State University, Columbus, Ohio, United States of America; Centre for Chronobiology, SWITZERLAND

## Abstract

Metabolic rate reduction has been considered the mechanism by which sleep conserves energy, similar to torpor or hibernation. This mechanism of energy savings is in conflict with the known upregulation (compared to wake) of diverse functions during sleep and neglects a potential role in energy conservation for partitioning of biological operations by behavioral state. Indeed, energy savings as derived from state-dependent resource allocations have yet to be examined. A mathematical model is presented based on relative rates of energy deployment for biological processes upregulated during either wake or sleep. Using this model, energy savings from sleep-wake cycling over constant wakefulness is computed by comparing stable limit cycles for systems of differential equations. A primary objective is to compare potential energy savings derived from state-dependent metabolic partitioning versus metabolic rate reduction. Additionally, energy conservation from sleep quota and the circadian system are also quantified in relation to a continuous wake condition. As a function of metabolic partitioning, our calculations show that coupling of metabolic operations with behavioral state may provide comparatively greater energy savings than the measured decrease in metabolic rate, suggesting that actual energy savings derived from sleep may be more than 4-fold greater than previous estimates. A combination of state-dependent metabolic partitioning and modest metabolic rate reduction during sleep may enhance energy savings beyond what is achievable through metabolic partitioning alone; however, the relative contribution from metabolic partitioning diminishes as metabolic rate is decreased during the rest phase. Sleep quota and the circadian system further augment energy savings in the model. Finally, we propose that state-dependent resource allocation underpins both sleep homeostasis and the optimization of daily energy conservation across species. This new paradigm identifies an evolutionary selective advantage for the upregulation of central and peripheral biological processes during sleep, presenting a unifying construct to understand sleep function.

## Introduction

Sleep has long been considered an energy conservation strategy similar to torpor or hibernation [1–3]. This current perspective views metabolic rate reduction during sleep as the mechanism of energy savings. However, fundamental shortcomings have prevented its broad acceptance. Contrary to a downregulation of biological processes anticipated by this theory, it is now well established that diverse functions become upregulated during sleep (compared to wake), establishing sleep as a highly active metabolic state. These upregulated functions in sleep include macromolecule biosynthesis, intracellular transport and membrane repair [4,5], neural network reorganization or memory consolidation [6–10], immune function [11] and restorative processes [12–14]. Moreover, the current energy conservation hypothesis has been criticized as providing only limited energy savings. To illustrate, an 8 h metabolic rate reduction of 15–30% during sleep compared to quiet wakefulness results in a 5–15% decrease in total daily (24 h) energy expenditure [15–17], a finding often cited as only a “cup of milk” for an adult human [16,17]. Such modest savings have raised skepticism that energy conservation is the universal function of sleep shared by all species [17–19], particularly given sleep’s inherent costs related to lost mating and foraging opportunities and increased predation risk from reduced behavioral responsiveness.

Previous calculations of energy savings derived from sleep are based only on metabolic rate reduction, a mathematical calculation that implicitly assumes all metabolic functions to be equally reduced during sleep compared to wake. However, the upregulation of many biological operations during sleep contradicts this assumption. Moreover, other processes are instead upregulated in wakefulness and downregulated in sleep, including excitatory neurotransmission, energy metabolism and responses to cellular stress [4,5]. These data are consistent with a state-dependent metabolic partitioning as outlined in the recently proposed energy allocation hypothesis of sleep [20]. This new paradigm postulates that state-dependent coupling of biological functions partitions energy resources in a manner that provides comparatively greater daily energy conservation than metabolic rate reduction [20]. Surprisingly, there are no published reports on energy savings derived from coupling biological processes with behavioral state.

Here we propose that actual energy savings from sleep may be more than 4-fold greater than previous estimates, primarily reflecting the contribution from state-dependent metabolic partitioning. Moreover, the model suggests that such partitioning is constrained if energy deployment toward waking-related processes is maintained during the rest phase, whereas energy savings is amplified if waking-related allocations during sleep are eliminated. Sleep quota and the circadian system further enhance energy conservation in the model. We conclude that the upregulation of central and peripheral biological processes during sleep is driven by the evolutionary selective advantage of partitioning metabolic operations, the principal mechanism by which sleep optimizes energy conservation across species.

## Results

A mathematical model is presented to quantify the relative contributions of metabolic partitioning, metabolic rate reduction during sleep (rho: ρ), sleep quota and the role of the circadian system in energy conservation. The amount of state-dependent metabolic partitioning is varied in the model using a metabolic allocation index (MAI). The MAI is quantified using the relative rates of energy deployment for biological functions directed toward either waking effort (r_W_) or biological investment (r_B_). Comparing an organism to a machine, r_W_ refers to the rate of energy deployed for “running” the machine (energy acquisition, predation avoidance and reproduction), whereas r_B_ to “maintenance” and “upgrading” of the machine. The model assumes that relative rates of energy deployment are state dependent with values of r_W_>0 and r_B_>0 permitted in both behavioral states. The MAI is considered to range from 0 to 1 to reflect the overall extent the “expected” processes are upregulated (r_W_ in wake, r_B_ in sleep).

**Fig 1:**
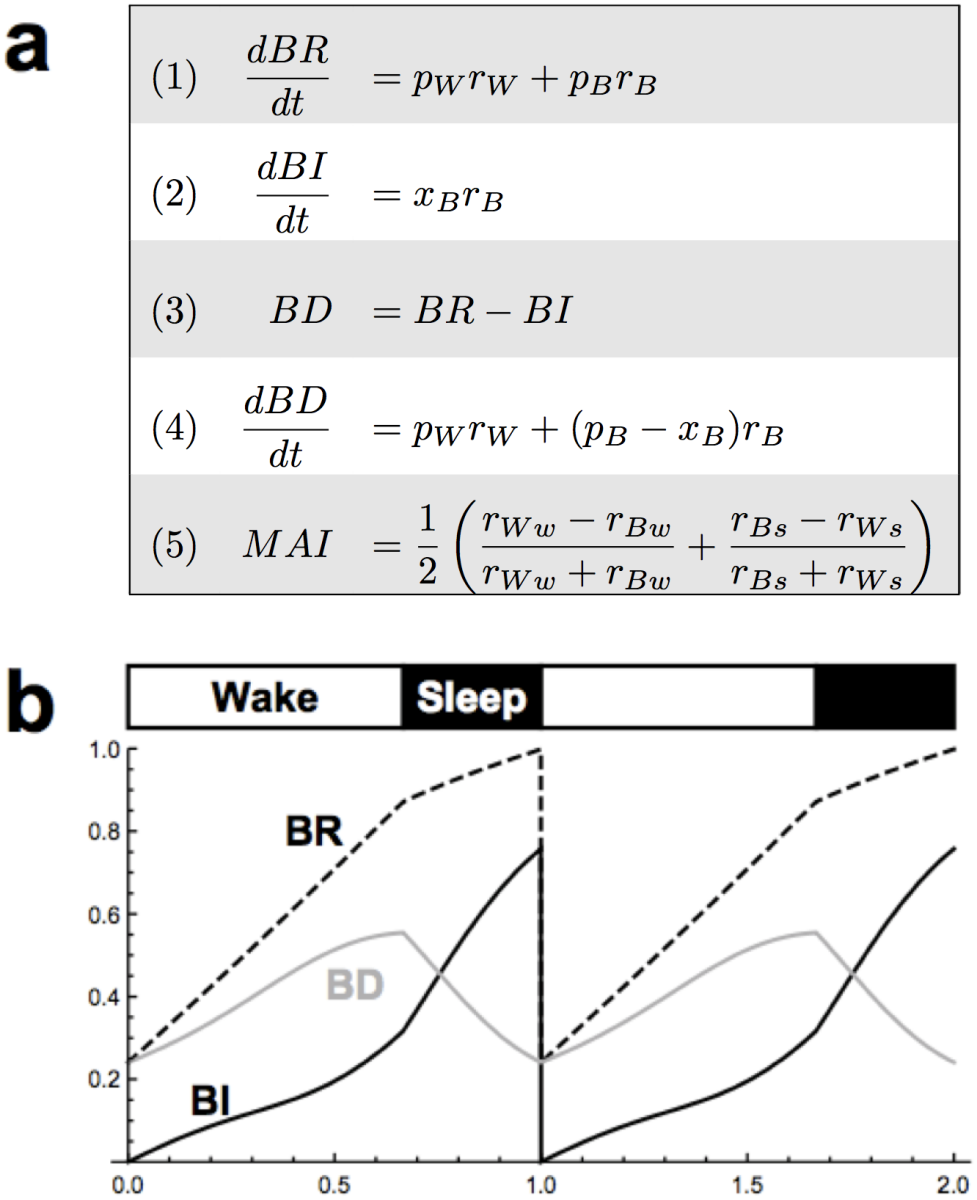
Equations and parameters. (A) Symbols r_W_ and r_B_ denote rates of energy to waking effort and biological investment (BI), respectively. Comparing an organism to a machine, r_W_ refers to the rate of energy deployed for “running” the machine (energy acquisition, predation avoidance and reproduction), whereas r_B_ to “maintenance” and “upgrading” of the machine. r_W_ and r_B_ contribute to growth in biological requirements (BR) dependent on their rates (equation 1), but only r_B_ is converted into BI (equation 2). Symbols p_W_ and p_B_ denote the “price” of expending energy on waking effort or BI, respectively. x_B_ denotes conversion of r_B_ to BI containing circadian and homeostatic components (see Methods). Biological debt (BD) is the difference between BR and BI (equation 3). Equations (1), (2), and (3) imply equation (4). Although r_W_ and r_B_ are constant within state, they differ among states, defining metabolic allocation index (MAI) in equation (5). A subscripted “w” or “s” denotes rates during wake or sleep, respectively, e.g., r_Ww_. (B) Solution to system of differential equations: At start of each day, BI is reset to 0, whereas BR is reset to the value of BD.

We assume that maintenance requirements are inherent to all biological systems if operational integrity is to be preserved. In our model, biological requirements (BR) represent the summation of maintenance obligations generated by all metabolic operations. Biological investments (BI), in contrast, are defined as the summation of completed functions servicing these requirements. As described in Fig 1, both r_W_ and r_B_ contribute to growth in biological requirements dependent on their rates, but only r_B_ is converted to biological investment. We also assume that natural selection favors organisms that manage energy expenditures while limiting biological debt. We thus track biological debt (BD) in arbitrary units by calculating at any given time the difference between biological requirements and investments (Fig 1b).

A basic premise of the model is that a state-dependent metabolic partitioning occurs at the whole organism level. That is, partitioning of metabolic operations is not restricted to a single organ or structure. MAI in the model defines the average partitioning of metabolic functions according to behavioral state across all organ systems. We hypothesize that such whole organism partitioning potentially increases energy savings beyond what a single organ system could otherwise achieve. Specific mechanisms are viewed to coordinate behavioral state (brain) with periphery, including state-specific hormonal release (anabolic in sleep, catabolic in wake), autonomic innervation of peripheral tissues, and synchronization of peripheral and central circadian clocks [20].

**Fig 2:**
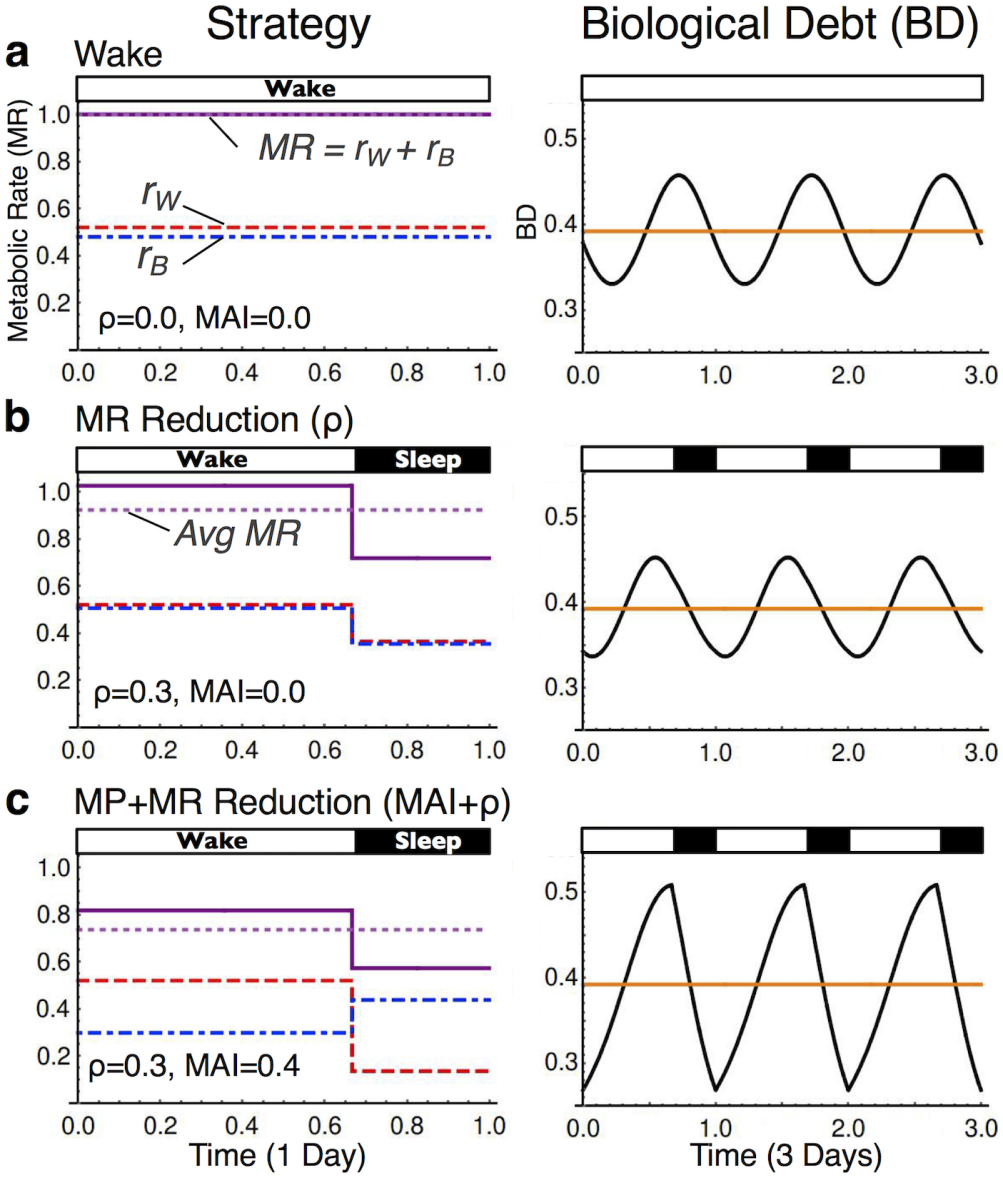
Method for calculating energy conservation. Three strategies are differentiated based on total sleep time (TST), metabolic rate (MR) reduction (ρ) during sleep, and state-dependent metabolic partitioning (MP) as defined by the metabolic allocation index (MAI). (A) Continuous wakefulness (TST = 0, ρ = 0 and MAI = 0) is the comparator state. (B) *Strategy MR Reduction (ρ)* cycles wake with sleep by introducing nonzero sleep quotas (TST>0) and ρ>0, while holding MAI = 0. (C) *Strategy MP+MR Reduction (MAI+ρ)* introduces MAI>0. In the two sleep conditions (B and C), r_W_ in wake is held constant with respect to *Strategy Wake* (A). Right panels show biological debt (BD) over three consecutive days at steady state. We impose the condition that daily average BD (orange) be held constant across conditions. To calculate energy savings, the value of r_B_ in wake is identified such that average BD matches continuous wakefulness (see Methods). Key: red (dashed) line is r_W_, blue (dot-dashed) line is r_B_, the dark purple (solid) line is metabolic rate (MR) such that MR = r_W_+r_B_, and the light purple (dotted) line is average MR. Standard parameters: p_W_ = 1.3, p_B1_ = 0.7, m_C_ = 5, A = 2.5.

Using the method demonstrated in Fig 2, continuous wakefulness is used as the comparator state for computations of energy savings derived from sleep. This continuous wakefulness condition (*Strategy Wake*, Fig 2a) is characterized mathematically by the absence of sleep (TST = 0 h), a constant metabolic rate (ρ = 0), and without partitioning of biological functions (MAI = 0). For the first sleep condition (*Strategy MR Reduction*, Fig 2b), energy savings from metabolic rate reduction (ρ) is calculated by introducing a TST>0 and ρ>0, while keeping MAI = 0 and waking effort (r_W_) constant with respect to the continuous wake strategy. We then compute the value of r_B_ in wake such that the average biological debt at steady-state matches that of continuous wakefulness (note that once r_B_ in wake is chosen, r_W_ and r_B_ in sleep are determined mathematically, see Methods). Average metabolic rate over one 24 h day is then compared to *Strategy Wake*, giving energy savings from metabolic rate reduction (ES_ρ_).

**Fig 3:**
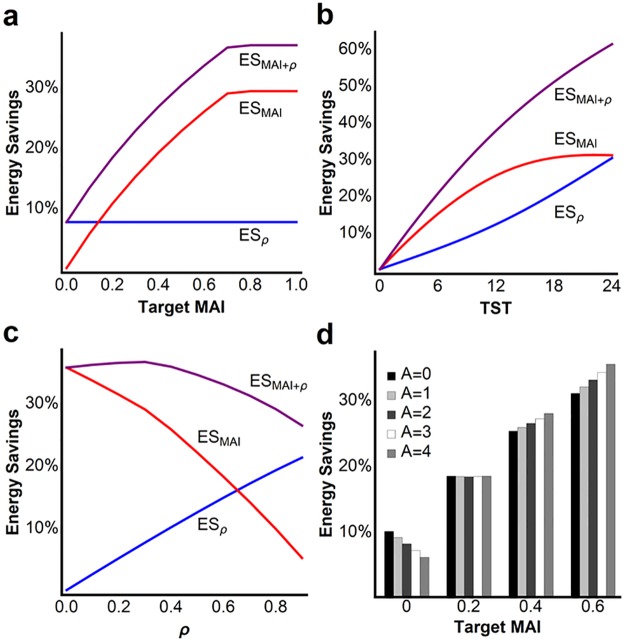
Energy savings calculations. (A) Target metabolic allocation index (MAI) is varied while holding metabolic rate reduction and TST constant. Note that an 8 h sleep quota and ρ = 0.3 in the figure constrain maximum MAI to ~0.7. (B) Varying TST with target MAI = 0.4 and ρ = 0.3. Gains in energy savings are primarily derived from MAI for TST<7 h or from ρ as TST exceeds 14 h. (C) Varying ρ with target MAI = 0.7 and TST = 8 h. Reductions in metabolic rate during the rest phase constrain MAI. Blue line is energy savings from ρ (ES_ρ_), red line is savings from MAI (ES_MAI_), and purple line is overall energy savings (ES_MAI+ρ_). (D) Energy savings as a function of target MAI and circadian amplitude (A) with ρ = 0.3 and TST = 8 h. Standard parameters and definitions utilized from Fig 1. Effects of variation in other model parameters on energy savings were low compared to effects of MAI, TST, ρ, and A (see S1 Methods and S3 Fig).

The above methodology demonstrates that a 30% reduction in metabolic rate (ρ = 0.3) for 8 h of sleep with MAI = 0 provides a 7.5% daily energy savings (ES_ρ_) over *Strategy Wake* (Fig 3a). This calculation is consistent with calorimetry data [15,17]. However, these published observations using calorimetry do not calculate energy savings derived from partitioning metabolic operations according to behavioral state (see below and Discussion).

For the second sleep strategy (*Strategy Metabolic Partitioning (MP)+MR Reduction*, Fig 2c), we additionally introduce state-dependent coupling of biological operations (MAI>0). Applying the methodology used above, a comparison with *Strategy Wake* gives overall energy savings (ES_MAI+ρ_) from both metabolic partitioning (MAI) and metabolic rate reduction (ρ). As shown in Fig 3a, maximizing MAI amplifies energy savings by approximately 4-fold over the alternative strategy of continuous wakefulness (Fig 3a), resulting in total energy savings of ~37% for an 8 h sleep quota. Note that, as illustrated in Fig 3a, a target MAI = 1 is not achievable under some combinations of parameters. For example, decreasing either sleep quota or metabolic rate during sleep constrains total achievable metabolic activity during the rest phase, forcing an increase in r_B_ during wake to service requirements and to limit a rise in average daily biological debt. Despite these constraints, energy savings from modest state-dependent metabolic partitioning equals or exceeds that from metabolic rate reduction across all sleep quotas (Fig 3b). Finally, the model predicts that metabolic rate reduction is not required for sleep to conserve energy. As demonstrated in Fig 3c, an 8 h sleep quota with a target MAI = 0.7 provides a calculated daily energy savings of 35% without reducing metabolic rate (ρ = 0).

The energy allocation model reveals unforeseen interactions between state-dependent metabolic partitioning and metabolic rate in energy conservation. For example, a combination of metabolic partitioning and metabolic rate reduction may enhance energy savings of sleep beyond what is achievable through metabolic partitioning alone (Fig 3c). However, the relative contribution from metabolic partitioning diminishes as ρ increases towards its maximum value of 1 (i.e., 100% metabolic rate reduction). Large increases in ρ, however, are more compatible with the behavioral state of torpor where reducing metabolic rate is the primary mechanism of energy savings [21,22].

We also identify an interaction between the circadian system and state-dependent metabolic partitioning in energy conservation. Circadian amplitude (A) provides additional daily energy savings in the presence of at least a moderate MAI (i.e., MAI≥0.4 in Fig 3d), reflecting an efficiency multiplier function of the circadian process in converting energy to biological investment in the model (see Methods). This circadian process is intended to model the role of central and peripheral molecular clocks in regulating metabolic functions at the local or cellular level [23–25]. In contrast, daily energy savings are reduced when circadian amplitude is increased in the absence of state-dependent metabolic partitioning (i.e., MAI = 0). This latter effect results from inefficiencies in expending energy on state-dependent processes when comparatively out of phase with the circadian system.

**Fig 4:**
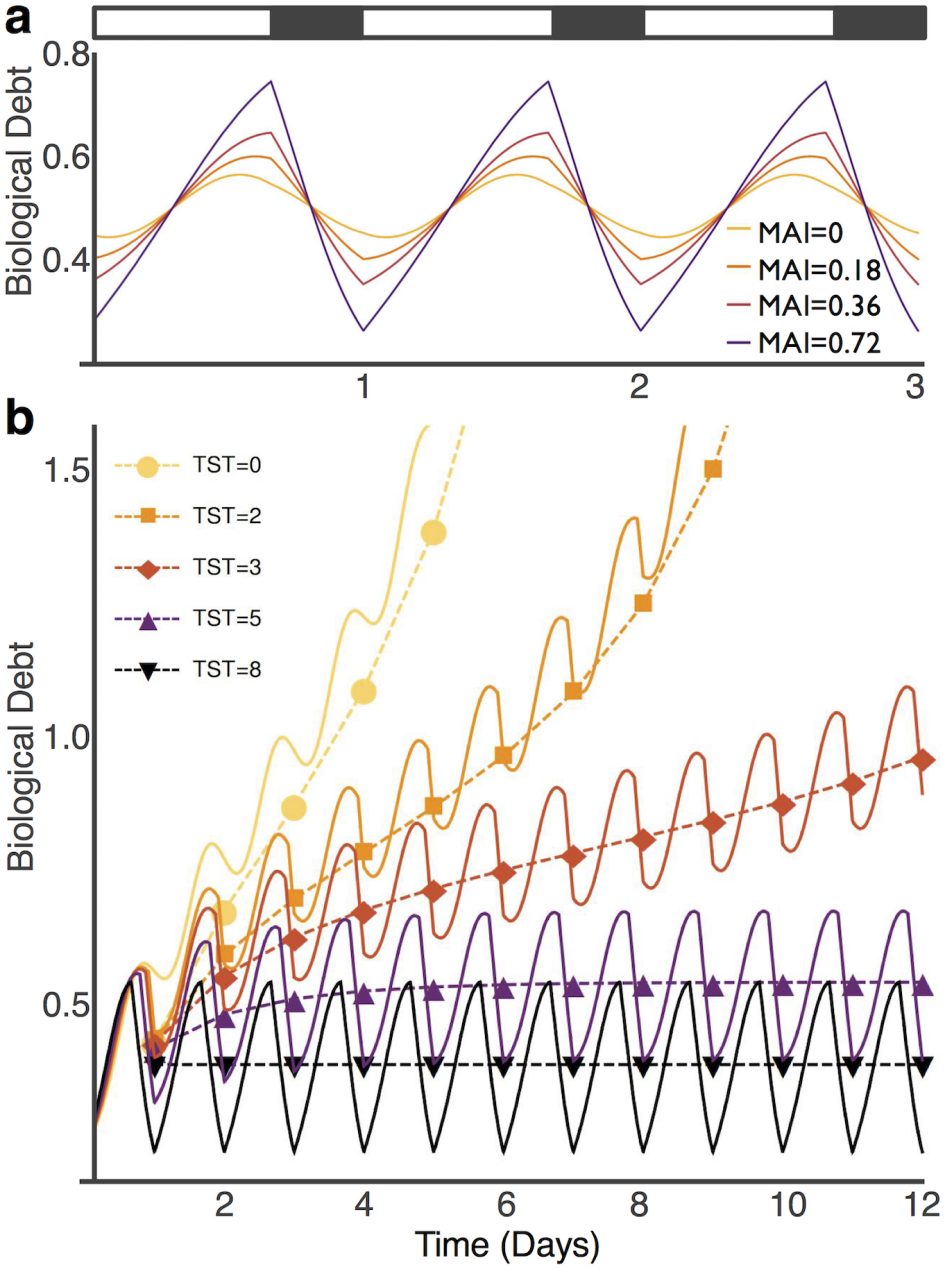
Temporal pattern of biological debt. (A) Biological debt (BD) in graphical form is modified by the metabolic allocation index (MAI). Specifically, the daily rise and fall of BD is transformed from a sinusoid when MAI = 0 and A>0 to an appearance resembling Process S in the two-process model [26] (25) when state-dependent metabolic partitioning (MAI>0) is introduced. (B) BD at varying levels of TST over 12 days. Dashed lines pass through average BD over the previous day. Small reductions in TST lead to elevations in average BD that may still attain steady state. However, rapid rises in mean BD are observed if r_B_ is not adequately increased to service biological requirements as daily TST approaches 0. This behavior of BD in the model is similar to empirical effects of sleep loss on human neurobehavioral performance (37). Steady-state is not reached when TST<4 h. Standard parameters as in Fig 1.

Monitoring of biological debt, while normalizing meaningful comparisons of energy savings calculations across strategies, demonstrates a well-defined temporal pattern that we postulate may impact sleep regulation. Biological debt in the model generally increases during wake and decreases during sleep, a behavior resulting from biological requirements exceeding investments during wake and investments predominating during sleep. Both the circadian system and state-dependent metabolic partitioning contribute to its temporal pattern. For example, provided steady state conditions are achieved for a given set of parameters, biological debt remains unchanging as a flat line over multiple days in the absence of both circadian amplitude (A = 0) and state-dependent metabolic partitioning (MAI = 0). However, the temporal pattern of biological debt becomes a circadian-influenced sinusoidal-like wave when A>0 in the absence of state-dependent metabolic partitioning (MAI = 0), reflecting circadian-dependent efficiencies in servicing biological requirements. Finally, biological debt is transformed from a sinusoidal wave into one that appears remarkably similar to Process S of the two-process model (see [26,27]) as MAI is introduced (Fig 4a), revealing nonlinear rises during wake and nonlinear declines during sleep.

Modeling over successive days with varying levels of sleep restriction reveals a temporal pattern of biological debt that resembles the behavior of Process S and its hypothesized role in homeostatic sleep regulation. For example, a small amount of sleep restriction results in a small increase in daily biological debt (Fig 4b), but the biological debt cycle remains stable at this new, elevated level. However, daily biological investment may not be sufficient to service requirements as daily sleep quota approaches 0 h, leading to rapid rises in mean biological debt (see Fig 4b) and an inability to achieve steady state, a condition we hypothesize may represent an “escape from homeostasis” [28].

## Discussion

The energy allocation model defines four variables that may impact energy savings derived from sleep. These include: 1) state-dependent metabolic partitioning (MAI), 2) metabolic rate reduction during sleep (ρ), 3) total sleep time (TST), and 4) circadian amplitude (A). In addition to quantifying the relative contributions of these variables in energy conservation, the model also identifies for the first time specific interactions between these variables potentially impacting energy savings. For example, metabolic rate typically decreases during the rest phase across species, including a 15–30% reduction during sleep [15,17] and more than 90% reduction during hibernation [21,22]. We show that although state-dependent metabolic partitioning has a greater potential impact on total energy savings when cycling between sleep and wakefulness, small reductions in metabolic rate during sleep may augment energy savings beyond what is achievable from metabolic partitioning alone. However, the relative contribution from metabolic partitioning diminishes as metabolic rate during the rest phase is reduced (Fig 3c). This interaction is consistent with the following proposition: State-dependent partitioning of metabolic processes is the primary mechanism of energy savings derived from sleep, whereas metabolic rate reduction the principle mechanism for torpor.

As a result of preferential coupling of unique biological functions with either sleep or wake, our calculations suggest that actual energy savings from sleep are potentially 4-fold greater than what was reported previously from metabolic rate reduction, theoretically reducing total energy requirements by over 50% for species with long sleep quotas (ES_MAI+ρ_, Fig 3b). We hypothesize that state-dependent metabolic partitioning occurs at the whole organism level, consistent with the great diversity of gene expression specifically coupled with either sleep or wakefulness in both central and peripheral tissues [4,5,29]. Whole organism energy expenditure influences an animal’s likelihood of overcoming energetic shortfall, accumulating energy reserves, and converting energy to offspring, thereby impacting its lifetime reproductive success. Our mathematical modeling suggests that the alternative strategy of continuous wakefulness increases biological requirements, constrains partitioning of metabolic processes, and would require greater metabolic investments to limit rises in biological debt. Moreover, performing all processes simultaneously (MAI = 0) theoretically increases cellular infrastructure requirements, an additional energy cost not addressed in the model. Experimental work is required to assess the role of coupling specific biological functions with either rapid eye movement (REM) or non-REM sleep to further exploit metabolic partitioning for energy conservation in endotherms as the energy allocation hypothesis postulates [20].

Prior work suggested that 8 h of sleep only reduces daily energy expenditures by 5–15% [15–17,30]. However, these previous observations, often employing short-term sleep restriction, are not designed to determine differences in net energy savings between habitual continuous wakefulness and habitual sleep-wake cycling. Specifically, we have identified two critical limitations of this prior work. First, mathematical calculations from these earlier studies rely on the implicit assumption that all biological processes are equally reduced during sleep compared to wake, not taking into consideration differences in resource allocations within each state. Indeed, our calculations of energy savings as derived only from metabolic rate reduction during sleep are consistent with this earlier work (see Fig 2b, Strategy MR reduction), a calculation that excludes contributions from metabolic partitioning. Second, the energy requirements to maintain comparable levels of biological debt when restricting sleep-dependent processes have not been considered. Our calculations suggest that daily energy requirements would be much greater for organisms to achieve habitual, long-term, continuous wakefulness while maintaining comparable levels of biological debt with respect to an alternative sleep-wake cycling strategy.

The proposed interactions in our model between metabolic partitioning, metabolic rate reduction, sleep quota, and the circadian system are not intuitively obvious, but they provide testable hypotheses on the optimization of sleep or torpor strategies employed across species for energy conservation. For example, sleep quotas vary widely by species from less than 4 h per day in some species to more than 18 h per day in others, a finding that remains unexplained and lacking clear significance with constitutive variables such as body mass [3,31–33]. For short sleepers (e.g., <4 h, Fig 3b), the effect of state-dependent metabolic partitioning (MAI) on energy savings is strong compared to that of metabolic rate reduction during sleep. Long sleep quotas, in contrast, achieve comparatively greater total energy savings, but the effect of metabolic partitioning on energy savings shows an asymptotic flattening as sleep quota exceeds 12–14 h while that of metabolic rate reduction continues to increase (see Fig 3b). These interactions predict short sleeping species to maintain a relatively elevated metabolic rate during sleep to optimize energy conservation through metabolic partitioning, a prediction requiring further investigation. Long-sleeping species, in contrast, should more likely reduce metabolic rate during sleep given its increasing gains toward energy savings as sleep quota approaches 24 h. Our review of the literature finds this latter prediction well supported: Species with habitual sleep quotas >12 h also commonly reduce metabolic rate by as much as 70% during the rest phase by entering daily (shallow) torpor periods (e.g., see species-family comparisons in [31,34,35]). Moreover, long sleeping endotherms are particularly prone to enter daily (nightly) torpor when challenged by energetic shortfalls [22,36,37], suggesting an adaptive capability to shift energy allocation strategies between primarily metabolic partitioning (sleep) or metabolic rate reduction (torpor) depending on energy status.

The energy allocation model also ascribes an important role for the circadian system in energy conservation when combined with at least a modest partitioning of metabolic operations by behavioral state (Fig 3d). However, if all biological processes are to be performed simultaneously without interruption (i.e., MAI = 0), the optimal strategy reducing daily energy expenditures is to dampen or eliminate circadian amplitude. This interaction between the circadian system and state-dependent metabolic partitioning is consistent with the close relationship between circadian and sleep-wake mechanisms. Circadian amplitude of gene expression, for example, is markedly reduced during sleep deprivation [38,39]. Sleep restriction in our model constrains the MAI by limiting biological investment during the rest phase. As a result, such investments must increase during extended wakefulness in the model (i.e., via increasing r_B_ during wake) to limit a rise in daily biological debt. Prolonged wakefulness, therefore, theoretically necessitates the organism perform a greater proportion of biological functions simultaneously. We view dampening of circadian amplitude during sleep loss as an adaptive response to more efficiently service biological requirements during prolonged waking bouts when state-dependent metabolic partitioning must be reduced.

Finally, we hypothesize that biological debt may ultimately govern sleep homeostasis. Biological systems that cyclically partition operations over time, as with either circadian-dependent or state-dependent coupling, face trade-offs with respect to such resource allocations. On the one hand, our model suggests that partitioning of operations conserves energy through efficiencies in resource utilization, a potential advantage for species influenced by the predictability of the Earth’s rotation and the daily cycling of its ecology. On the other hand, biological debt will rise if sleep-dependent processes are restricted through sleep deprivation.

Process S in the two-process model was a major advancement in understanding the homeostatic drive for sleep [26,27], even though the underlying basis for its behavior remains unknown. For example, no somnogenic factor accumulating during wake and dissipating during sleep has been identified adequately resembling its temporal rise and fall. The energy allocation model demonstrates that the daily variation in biological debt resembles Process S of the two-process model with respect to its nonlinear rise in wake and fall in sleep, a specific morphological pattern that only appears in our model when state-dependent coupling of biological functions (MAI) is introduced (see Fig 4a). Moreover, small reductions in TST lead to small but stable daily elevations in biological debt, whereas more significant reductions of sleep may lead to sudden, nonlinear, escalations over multiple days, theoretically reflecting an escape from homeostasis [28] (see Fig 4a). This behavior of biological debt models empirical effects of sleep loss on human neurobehavioral performance and is consistent with homeostatic sleep propensity [26,27,40]. The mechanistic link, however, between biological debt and known signals of homeostatic sleep pressure, such as extracellular adenosine [41,42], remains to be elucidated.

Given the calculated impact of sleep-wake cycling on reducing daily energy requirements, we propose that the ultimate (evolutionary) function of sleep is energy conservation through a state-dependent coupling of biological operations. We suggest that basic principles of this general model may be applicable to all species, potentially providing insight into one of biology’s greatest questions: What is the selective advantage of sleep over the alternative behavioral strategy of quiet wakefulness? The answer, we propose, resides in state-dependent metabolic partitioning, a mechanism that amplifies energy savings as waking-related allocations are eliminated during a metabolically active rest phase where unique biological processes are upregulated. This unifying perspective does not conflict with the many proposed physiological (proximate) functions of sleep, including the upregulation of protein biosynthesis, immune function, neural network reorganization and restoration. On the contrary, the upregulation of these diverse functions during sleep is viewed to be in the service of state-dependent metabolic partitioning, a mechanism by which daily energy conservation is optimized.

## Methods

We assume the presence of two monophasic, consolidated states. The total time spent in the two states is one day (24 hours), beginning with wake (0 < *t* < 1 − TST/24) and followed by sleep (1 − TST/24 < *t* < 1). These states may differ by their allocations of energy to processes according to the strategy employed.

We model BR, BI, and BD with differential equations defined in Fig 1a. We further define

$$p_{B}\left(t\right)=p_{B1}\text{BD}\left(t\right),$$

so that the price of BI is proportional to BD. We also define

$$x_{B}\left(t\right)=C\left(t\right)\frac{\text{BD}\left(t\right)}{1+\text{BD}{\left(t\right)}^{2}},$$

where *C*(*t*) is defined

$$C\left(t\right)=m_{C}-A\text{sin}\left(2\pi \left(t-0.25+0.5\left(\frac{\text{TST}}{24}\right)\right)\right).$$

The argument of sine in the above equation was chosen for the period to be one day (24 hours) and so that the peak occurs in the middle of the sleep phase. We require all parameters to be nonnegative. Additionally, BD > 0, *r*_*W*_ ≥ 0 and *r*_*B*_ ≥ 0, and *A* < *m*_*C*_ (so that *C* is positive). See Fig 1b for a graph of solutions for BR, BI, and BD.

The conversion factor *x*_*B*_(*t*) contains two independent efficiency multipliers. First, the circadian process, *C*(*t*), participates as an efficiency multiplier in the conversion of energy (*r*_*B*_) to BI, providing greater efficiency during the sleep phase and less during the wake phase. Circadian amplitude (*A*) is half the peak to trough range. This circadian model reflects the assumed role of circadian molecular clocks in regulating metabolic processes at the local or cellular level. The circadian system in the EA model, therefore, contributes to the shape of the BD curve (see Figs 1b and 4a). The second efficiency multiplier component of *x*_*B*_(*t*) depends on the level of BD to model a reactive homeostasis in the conversion of energy to BI. The curve of this reactive homeostatic component is low at low levels of BD, peaks at some moderate level of BD, and decreases asymptotically to 0 as BD increases; this shape reflects greatest efficiency in energy conversion at some moderate level of BD but with decreasing efficiency at either low or high levels of BD.

When computing energy savings, we attempt to keep average BD the same for each strategy. When a periodic solution exists, we interpret "average BD" (*m*_*BD*_) as the average BD over one period (one day). By considering Poincaré maps, we see that there are bifurcations of limit cycles, or periodic solutions (see S1 Methods). We consider the average BD of (half-) stable periodic solutions. We disregard cases in which there are no periodic solutions.

### Energy savings computation

Derived parameters *ρ* and MAI are useful for quantifying concepts in the EA model. Parameter *ρ* is the proportion of metabolic rate (MR) reduction from wake to sleep:

$$\rho =\frac{{\text{MR}}_{w}-{\text{MR}}_{s}}{{\text{MR}}_{w}}=1-\frac{r_{Ws}+r_{Bs}}{r_{Ww}+r_{Bw}}.$$

Parameter *ρ* may range from 0 to 1, where *ρ* = 0 means no MR reduction, and *ρ* = 1 means 100% MR reduction (MR_*s*_ = 0).

The metabolic allocation index parameter is defined

$$\text{MAI}=\frac{1}{2}\left(\frac{r_{Ww}-r_{Bw}}{r_{Ww}+r_{Bw}}+\frac{r_{Bs}-r_{Ws}}{r_{Ws}+r_{Bs}}\right)$$

MAI may theoretically take values between -1 and 1, but we consider values ranging between 0 and 1 to reflect the extent to which the "expected" processes are upregulated. Note that MAI = 0 means that the energy allocations are the same in both wake and sleep (i.e. $\frac{r_{Ww}}{r_{Bw}}=\frac{r_{Ws}}{r_{Bs}}$), while MAI = 1 implies *r*_*Bw*_ = *r*_*Ws*_ = 0.

To compute energy savings, we compare three strategies: *Strategy Wake*, *Strategy MR Reduction*, and *Strategy metabolic partitioning (MP) + MR Reduction*. See Results and Fig 2 for definitions. In order to compute energy savings, we must find the four energy rates (*r*_*Ww*_, *r*_*Bw*_, *r*_*Ws*_, *r*_*Bs*_) subject to conditions that are strategy-dependent. For *Strategy Wake* (TST = 0), we choose *r*_*Ww*_ = *r*_*Bw*_. Using the stable limit cycle solution to the differential equation, we compute *m*_BD_. We then compute the average metabolic rate *m*_*MR*1_ = *r*_*Ww*_ + *r*_*Bw*_.

For the other two strategies, we seek values for the four energy rates such the following criteria are satisfied: 1) *ρ* equals its chosen value, 2) *r*_*Ww*_ is maintained across strategies, 3) *m*_*BD*_ is maintained across conditions, and 4) MAI equals its chosen value. While we always meet the first two criteria, we may not always be able to satisfy the last two. To see this, we define our four energy rates as follows: *r*_*Ww*_ is constant, *r*_*Bw*_ is variable, and the remaining two rates, *r*_*Ws*_ and *r*_*Bs*_ are functions of *r*_*Bw*_, *ρ*, and MAI. Solving the equations for *ρ* and MAI explicitly, we have

$$\begin{array}{ll} r_{Ws}\left(r_{Bw},\rho ,\text{MAI}\right) & =\text{max}\left\{0,\left(1-\rho \right)\left(\left(1-\text{MAI}\right)r_{Ww}-\left(\text{MAI}\right)r_{Bw}\right)\right\}\\ r_{Bs}\left(r_{Bw},\rho ,\text{MAI}\right) & =\left(1-\rho \right)\left(r_{Ww}+r_{Bw}\right)-r_{Ws}, \end{array}$$

Note that these equations are defined so that all energy rates are positive. This restriction implies that condition (4) may not be able to be met. For reasons that condition (3) may not be able to be met, see S1 Methods.

To find values for the energy rates that will equalize *m*_*BD*_ between *Strategy Wake* and the other strategies, we adjust *r*_*Bw*_ starting at *r*_*Bw*_ = 0 and increase it until we reach the same *m*_*BD*_ as in *Strategy Wake*. The values of *r*_*Ws*_ and *r*_*Bs*_ are determined by chosen *ρ* and MAI, as seen above. For *Strategy MR Reduction*, *ρ* > 0 and MAI = 0. For *Strategy MP + MR Reduction* we use the same value of *ρ* and a chosen value of MAI > 0. We then compute *m*_*MR*2_ (the average metabolic rate for *Strategy MR Reduction*) and *m*_*MR*3_ (the average metabolic rate for *Strategy MP + MR Reduction*). Average metabolic rate is defined

$$m_{MR}=\left(1-\frac{\text{TST}}{24}\right)\left(r_{Ww}+r_{Bw}\right)+\left(\frac{\text{TST}}{24}\right)\left(r_{Ws}+r_{Bs}\right).$$

Adjusting the *r*_*Bw*_, *r*_*Ws*_, and *r*_*Bs*_ in this manner may not always provide the same *m*_*BD*_ across the three different strategies. Occasionally, it is only possible for average BD for one or both of the sleep strategies to be less than *m*_BD_ for *Strategy Wake* (see S1 Methods).

Energy savings from *ρ* and MAI may then be computed in the following manner: 1) Energy savings from *ρ* is *ES*_*ρ*_ = (*m*_*MR*1_ − *m*_*MR*2_)/*m*_*MR*1_, 2) overall energy savings from MAI and *ρ* is ES_MAI+*ρ*_ = (*m*_*MR*1_ − *m*_*MR*3_)/*m*_*MR*1_, and 3) energy savings from MAI is *ES*_MAI_ = *ES*_MAI+*ρ*_ − *ES*_*ρ*_.

## Supporting information

S1 Fig Energy allocation and MAI. *r*_*W*_ is the dashed red line, *r*_*B*_ is dot-dashed blue line, and MR is solid purple line. While calculating energy savings, we attempt to reach a target MAI of 0.6 with *ρ* = 0.3. In (A), (B), and (C), this target MAI is achieved while *r*_*Bw*_ is being increased and *r*_*Ws*_ is being decreased in response. In (D), we see that if *r*_*Bw*_ is increased further, MAI will be constrained since *r*_*Ws*_ must be non-negative. Thus, the target MAI of 0.6 cannot be met and MAI is reduced to 0.510 in this example. (Parameters: TST = 8 h, *r*_*Ww*_ = 0.5, (A) *r*_*Bw*_ = 0, (B) *r*_*Bw*_ = 0.2, (C) *r*_*Bw*_ = 0.33, (D) *r*_*Bw*_ = 0.48).
(TIFF)

S2 Fig Energy savings and the Poincaré map. Horizontal x-axis is BD at the beginning of the day, whereas the vertical y-axis is BD at the end of the day. The blue curve is the Poincaré map, the black line is the line *y* = *x*, the red line is *m*_BD_ of *Strategy Wake*, and the green line is the average BD of the alternative sleep-wake strategy. Fixed points of the Poincaré map occur when the blue curve intersects the black line. (Standard parameters: *p*_*W*_ = 1.3, *p*_*B*1_ = 0.7, *m*_*C*_ = 5, *A* = 2.5, *r*_*Ww*_ = 0.5). (A) *Strategy Wake*, MAI = 0 and *ρ* = 0. Stable Poincaré fixed point at BD ≈ 0.336. (Parameters: TST = 0 h, *r*_*Ww*_ = *r*_*Bw*_ = *r*_*Ws*_ = *r*_*Bs*_ = 0.5). (B) *Strategy MP + MR Reduction*, zero fixed points of Poincaré map. In this and remaining panels, MAI = 0.4 and *ρ* = 0.3 (Parameters: TST = 8 h, *r*_*Ww*_ = 0.5, *r*_*Bw*_ = 0, *r*_*Ws*_ = 0.21, *r*_*Bs*_ = 0.14). (C) *Strategy MP + MR Reduction*, one fixed point of Poincaré map. The bifurcation occurs, and a limit cycle comes into existence. The average BD of this limit cycle is shown in green and exceeds *m*_BD_ of *Strategy Wake* shown in red. If instead, the green line is below the red line at the bifurcation point, we compute energy savings at that point. (Parameters: TST = 8 h, *r*_*Ww*_ = 0.5, *r*_*Bw*_ = 0.20079, *r*_*Ws*_ = 0.15378, *r*_*Bs*_ = 0.33678). (D) *Strategy MP + MR Reduction*, two fixed points of Poincaré map. The average BD is the same as *Strategy Wake*. Energy savings is computed at this stage; here, ES_MAI+*ρ*_ ≈ 28%. (Parameters: TST = 8 h, *r*_*Ww*_ = 0.5, *r*_*Bw*_ = 0.3, *r*_*Ws*_ = 0.126, *r*_*Bs*_ = 0.434).
(TIFF)

S3 Fig Low sensitivity of parameters *p*_*W*_, *p*_*B*1_, and *m*_*C*_ was observed for energy savings calculations. Each box-and-whisker plot has a different fixed value of *m*_*C*_, with values of energy savings resulting from varying *p*_*W*_ and *p*_*B*1_ from 0 to 2 in intervals of 0.1 (if the system has a limit cycle). At any given value of *m*_*C*_, varying *p*_*W*_ and *p*_*B*1_ over a wide range of values accounts for only a 1–4% change in energy savings. Varying *m*_*C*_ in addition to *p*_*W*_ and *p*_*B*1_ accounts for a total range of energy savings of less than 8%. The middle vertical bar in each box-and-whisker plot represents the median value, the box is one quartile on either side of the median, and the whiskers represent the lowest and highest quartiles of values. (Parameters: TST = 8 h, *ρ* = 0.3, target MAI = 0.4, *A* = 0.5 * *m*_*C*_).
(TIFF)

S1 Methods Additional supporting information for methods. (DOCX)
