# Supplementary figures and images for: State-dependent metabolic partitioning and energy conservation: A theoretical framework for understanding the function of sleep
Source: PLoS One. 2017 Oct 10;12(10):e0185746. doi: 10.1371/journal.pone.0185746 (PMC5634544; doi:10.1371/journal.pone.0185746)

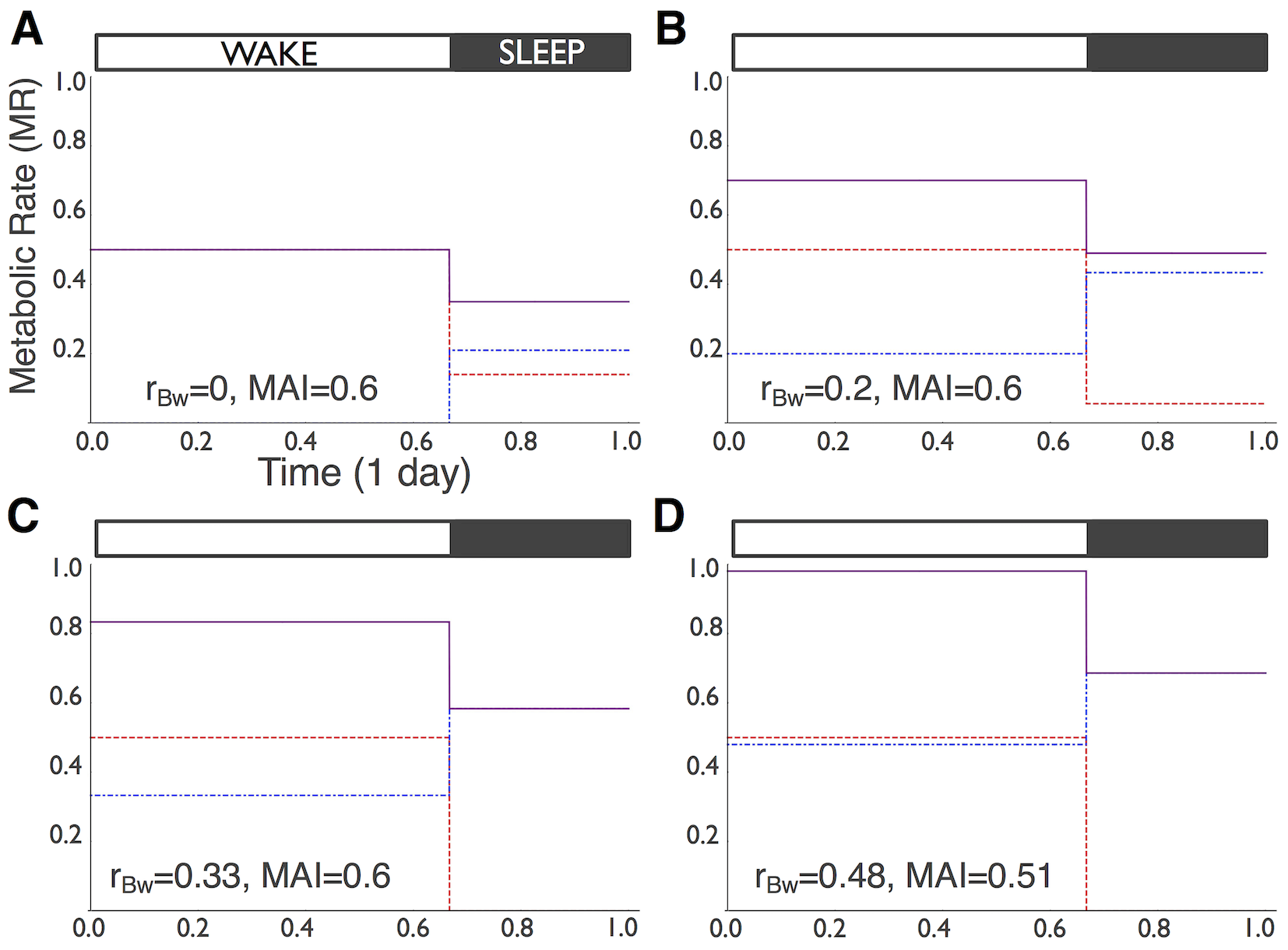

Supplement: S1 Fig — rW is the dashed red line, rB is dot-dashed blue line, and MR is solid purple line. While calculating energy savings, we attempt to reach a target MAI of 0.6 with ρ = 0.3. In (A), (B), and (C), this target MAI is achieved while rBw is being increased and rWs is being decreased in response. In (D), we see that if rBw is increased further, MAI will be constrained since rWs must be non-negative. Thus, the target MAI of 0.6 cannot be met and MAI is reduced to 0.510 in this example. (Parameters: TST = 8 h, rWw = 0.5, (A) rBw = 0, (B) rBw = 0.2, (C) rBw = 0.33, (D) rBw = 0.48). (TIFF) [file pone.0185746.s001.tiff]

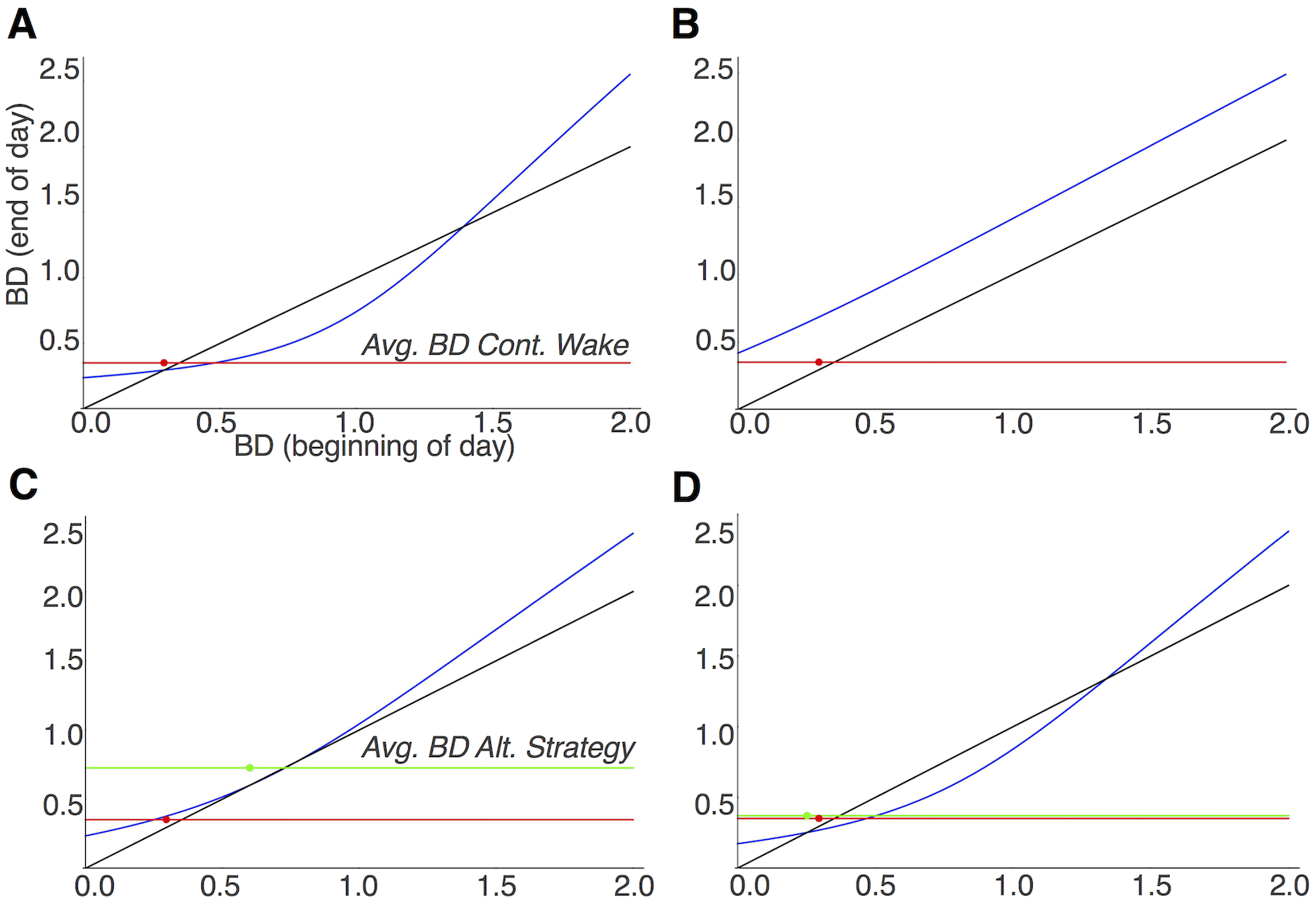

Supplement: S2 Fig — Horizontal x-axis is BD at the beginning of the day, whereas the vertical y-axis is BD at the end of the day. The blue curve is the Poincaré map, the black line is the line y = x, the red line is mBD of Strategy Wake, and the green line is the average BD of the alternative sleep-wake strategy. Fixed points of the Poincaré map occur when the blue curve intersects the black line. (Standard parameters: pW = 1.3, pB1 = 0.7, mC = 5, A = 2.5, rWw = 0.5). (A) Strategy Wake, MAI = 0 and ρ = 0. Stable Poincaré fixed point at BD ≈ 0.336. (Parameters: TST = 0 h, rWw = rBw = rWs = rBs = 0.5). (B) Strategy MP + MR Reduction, zero fixed points of Poincaré map. In this and remaining panels, MAI = 0.4 and ρ = 0.3 (Parameters: TST = 8 h, rWw = 0.5, rBw = 0, rWs = 0.21, rBs = 0.14). (C) Strategy MP + MR Reduction, one fixed point of Poincaré map. The bifurcation occurs, and a limit cycle comes into existence. The average BD of this limit cycle is shown in green and exceeds mBD of Strategy Wake shown in red. If instead, the green line is below the red line at the bifurcation point, we compute energy savings at that point. (Parameters: TST = 8 h, rWw = 0.5, rBw = 0.20079, rWs = 0.15378, rBs = 0.33678). (D) Strategy MP + MR Reduction, two fixed points of Poincaré map. The average BD is the same as Strategy Wake. Energy savings is computed at this stage; here, ESMAI+ρ ≈ 28%. (Parameters: TST = 8 h, rWw = 0.5, rBw = 0.3, rWs = 0.126, rBs = 0.434). (TIFF) [file pone.0185746.s002.tiff]

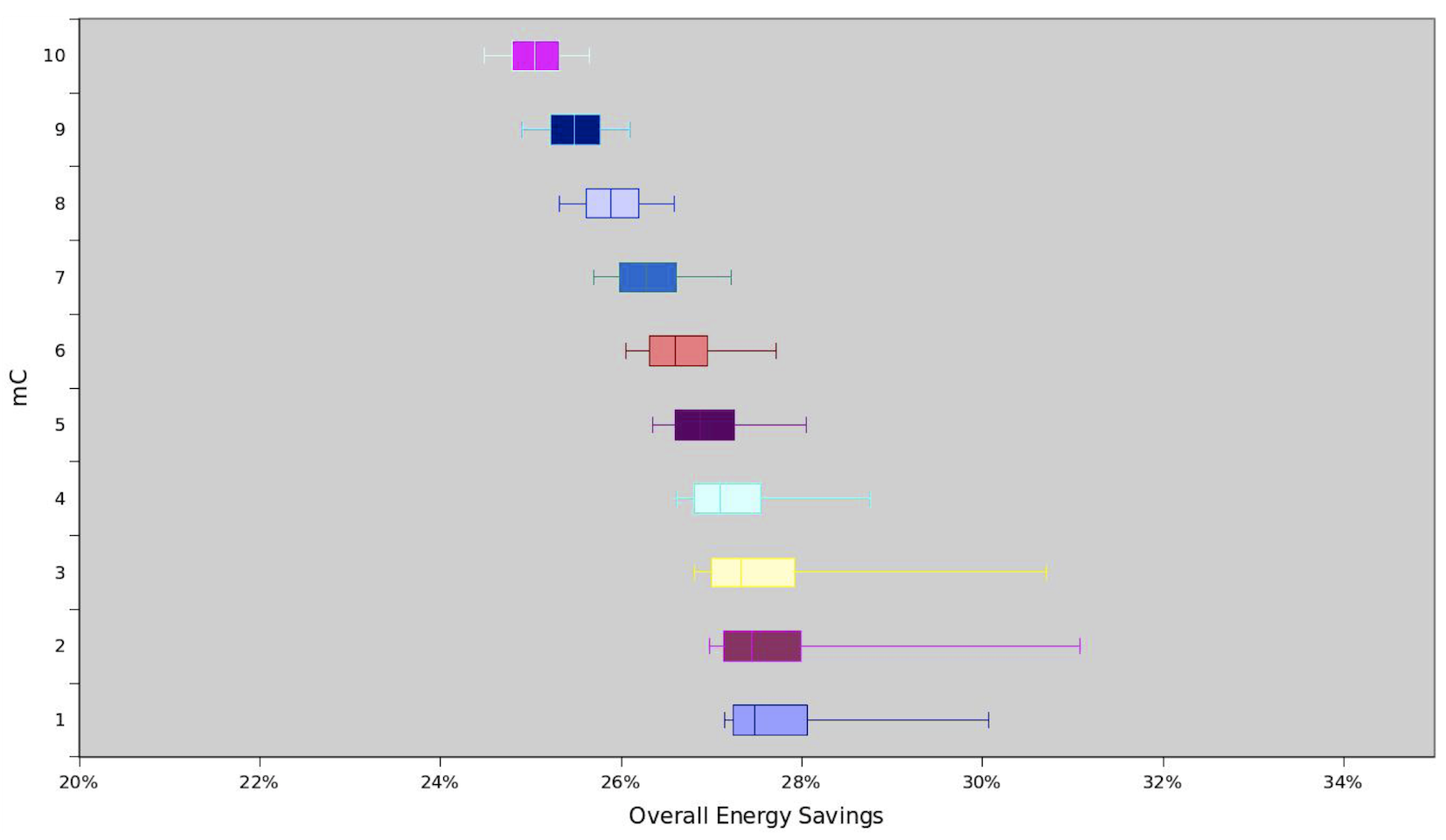

Supplement: S3 Fig — Each box-and-whisker plot has a different fixed value of mC, with values of energy savings resulting from varying pW and pB1 from 0 to 2 in intervals of 0.1 (if the system has a limit cycle). At any given value of mC, varying pW and pB1 over a wide range of values accounts for only a 1–4% change in energy savings. Varying mC in addition to pW and pB1 accounts for a total range of energy savings of less than 8%. The middle vertical bar in each box-and-whisker plot represents the median value, the box is one quartile on either side of the median, and the whiskers represent the lowest and highest quartiles of values. (Parameters: TST = 8 h, ρ = 0.3, target MAI = 0.4, A = 0.5 * mC). (TIFF) [file pone.0185746.s003.tiff]
