# Supplementary material for: State-dependent metabolic partitioning and energy conservation: A theoretical framework for understanding the function of sleep
Source: PLoS One. 2017 Oct 10;12(10):e0185746. doi: 10.1371/journal.pone.0185746 (PMC5634544; doi:10.1371/journal.pone.0185746)
Supplement: S1 Methods — (DOCX) [file pone.0185746.s004.docx]

Supporting Methods for:

State-Dependent Metabolic Partitioning and Energy Conservation: A Theoretical Framework for Understanding the Function of Sleep

Markus H. Schmidt*^1,2^, Theodore W. Swang^3^, Ian M. Hamilton^4^, and Janet A. Best^3^

^1^ Department of Neurology, University of Bern, Inselspital, Bern, Switzerland.

^2^Ohio Sleep Medicine and Neuroscience Institute, 4975 Bradenton Ave., Dublin, Ohio, USA.

^3^Department of Mathematics, The Ohio State University, Columbus OH, USA.

^4^Department of Evolution, Ecology and Organismal Biology, Department of Mathematics, The Ohio State University, Columbus OH, USA.

*Corresponding author: Department of Neurology, University of Bern, Inselspital, 3010 Bern, Switzerland, Email: [mschmidt@sleepmedicine.com](mailto:mschmidt@sleepmedicine.com), Tel: +41 78 895 71 13.

$${}{}$$

$${}\frac{}{{}}$$

$$\begin{matrix} {}\left( {} \right) & \left\{ \left( {}\left( \right){} \right) \right\} \\ {}\left( {} \right) & {}{}{} \end{matrix}$$

$${}\left( \frac{}{} \right){}{}\left( \frac{}{} \right){}{}$$

**Supporting Methods**

**Target** $\mathbf{MAI}$

Because the energy rates are nonnegative and $r_{Ww}$ is fixed across strategies, a $\mathrm{MAI}$ chosen before the calculation may not always be achievable if $m_{\mathrm{BD}}$ is to be fixed. For this reason, we call the value of $\mathrm{MAI}$ chosen prior to the calculation "target $\mathrm{MAI}$." For example, suppose the target $\mathrm{MAI}$ is 1, which can only happen when $r_{Bw}=r_{Ws}=0$. If this condition results in a value of $m_{\mathrm{BD}}$ greater than *Strategy Wake*, we increase $r_{Bw}$ (and do not let $r_{Ws}$ fall below 0) until the $m_{\mathrm{BD}}$ of *Strategy Wake* is met. This necessarily decreases overall $\mathrm{MAI}$ (see S1 Fig for another example). In this way, the method prioritizes $m_{\mathrm{BD}}$ over $\mathrm{MAI}$. This problem only applies to large values of target $\mathrm{MAI}$; the actual values of $\mathrm{MAI}$ resulting from the calculation are the maximum possible that also satisfy $m_{\mathrm{BD}}$ being held constant across strategies. We have also identified other conditions that may constrain $\mathrm{MAI}$ and prevent a target $\mathrm{MAI}$ from being achieved. These include decreasing $\mathrm{TST}$, decreasing circadian amplitude ($A$), or increasing $\rho$. These three examples will limit $r_{Bs}$ and require an increase in $r_{Bw}$ if $m_{\mathrm{BD}}$ is to be maintained.

**Poincaré Maps**

We record here, for convenience, the one-dimensional non-autonomous differential equation that $\mathrm{BD}$ satisfies:

$$\frac{d\mathrm{BD}}{dt}=p_{W}r_{W}(t)+p_{B1}r_{B}(t)\mathrm{BD}(t)-r_{B}(t)C(t)\frac{\mathrm{BD}(t)}{1+\mathrm{BD}(t)^{2}},$$

where $C$ is defined in the methods section.

Let $\mathrm{BD}(t;b_{0})$ denote the solution to the above differential equation with initial condition $\mathrm{BD}(0)=b_{0}>0$. We define the Poincaré map $P(z)=\mathrm{BD}(1;z)$.

For $z>0$, numerical evidence suggests that $P$ undergoes a saddle-node bifurcation, having either 0 fixed points, 1 fixed point (half-stable), or 2 fixed points (one stable, $z_{s}$; one unstable, $z_{u}$; with $z_{s}<z_{u}$). In the case of 2 fixed points, $\mathrm{BD}$ has one stable and one unstable limit cycle. The solution to the differential equation will approach the stable limit cycle unless $b_{0}>z_{u}$. In the case of 1 fixed point, $\mathrm{BD}$ has one half-stable limit cycle. The solution to the differential equation will approach the limit cycle unless $b_{0}$ is greater than the fixed point. In the case of 0 fixed points, no limit cycle exists, and $\mathrm{BD}$ will increase without bound. For examples of each of these cases, see S2 Fig b-d.

Adjusting $r_{Bw}$ (while simultaneously adjusting $r_{Ws}$ and $r_{Bs}$ as described in the methods section) may sometimes cause a bifurcation in which a stable limit cycle comes into existence (S2 Fig). Increasing $r_{Bw}$ while keeping $\rho$ and $\mathrm{MAI}$ fixed only decreases the stable fixed point of the Poincaré map, so if the stable limit cycle comes into existence with an average $\mathrm{BD}$ lower than $m_{BD}$ of the *Strategy Wake*, it may be impossible to achieve the same $m_{\mathrm{BD}}$. When this happens, we stop increasing $r_{Bw}$ when the limit cycle comes into existence, and record the average metabolic rate at that point.

**Parameter Sensitivity**

We calculated energy savings for over 243,000 sets of parameters. We found that the main effects of energy savings come from varying $\mathrm{MAI}$, $\rho$, $\mathrm{TST}$, and $A$. In cases we examined, parameters $p_{W}$, $p_{B1}$, and $m_{C}$ had only a minor effect on energy savings. This is not surprising, as these three parameters were held constant across conditions. A slightly more surprising result was the effect of the parameter $A$ on energy savings. Even though $A$ was held constant across conditions, the effect of $A$ on energy savings was conditioned on $\rho$ and $\mathrm{MAI}$. At low values of $\mathrm{MAI}$, increasing $A$ caused a drop in energy savings, while at high $\mathrm{MAI}$, increasing $A$ caused an increase in energy savings (Fig 3d in the main text). We saw the reverse effect with $\rho$; at low values of $\rho$, increasing $A$ increased energy savings, while at high values of $\rho$, increasing $A$ caused energy savings to decrease.

To measure the sensitivity of the parameters $p_{W}$, $p_{B1}$, and $m_{C}$, we varied $p_{W}$ and $p_{B1}$ with values ranging from 0.1 to 2 in increments of 0.1, and we varied $m_{C}$ with values ranging from 1 to 10 in increments of 1 (typically, for a system to reach steady state, $m_{C}$ must be large compared to $p_{W}$ and $p_{B1}$). While varying these parameters, we also varied $\rho$, $\mathrm{MAI}$, and $\mathrm{TST}$ separately. In each case, we saw that varying $\rho$, $\mathrm{MAI}$, or $\mathrm{TST}$ had a major effect on energy savings. However, within each fixed value of $\rho$, $\mathrm{MAI}$, or $\mathrm{TST}$, varying $p_{W}$, $p_{B1}$, and $m_{C}$, had only minor effects on energy savings (S3 Fig).

**Strategy Wake and** $\mathbf{MAI}$

We consider *Strategy Wake* to be a strategy of continuous wakefulness. In our calculation, we take $r_{Ww}=r_{Bw}$ under the assumption of zero $\mathrm{MAI}$ (because $\mathrm{TST}=0$, the values of $r_{Ws}$ and $r_{Bs}$ are not defined). However, it is possible to have $r_{Bw}\neq r_{Ww}$ in a continuous waking strategy. Note that continuous wakefulness ($\mathrm{TST}=0$) is equivalent to the strategy with $\mathrm{TST}>0$, $\rho=0$, and $\mathrm{MAI}=0$, as the last two conditions imply $r_{Ww}=r_{Ws}$ and $r_{Bw}=r_{Bs}$. In this way, we can still impose the condition $\mathrm{MAI}=0$ without having $r_{Bw}=r_{Ww}$. There is little impact on energy savings if these two rates are not equal for *Strategy Wake*.
